# Investigating the effect of pharmaceutical logistics service performance on customer satisfaction: a two-step approach with structural equation modeling

**DOI:** 10.1186/s40545-021-00351-6

**Published:** 2021-08-02

**Authors:** Tafesse Gizaw, Mekonnen Bogale, Tadesse Gudeta

**Affiliations:** 1 College of Business and Economics, Jimma University, Jimma, Ethiopia; 2 School of Pharmacy, Faculty of Health Sciences, Jimma University, Jimma, Ethiopia

**Keywords:** Pharmaceutical logistics service, Customer satisfaction, Structural Equation Modelling, Public health facilities, Ethiopian Pharmaceutical Supply Agency

## Abstract

**Background:**

These days, pharmaceutical customers are the utmost stakeholders in the healthcare supply chain, and ensuring their satisfaction with the logistics services has become worthwhile. This study aimed to investigate the effect of perceived logistics service performance on customer satisfaction in the public health facilities served under the Ethiopian Pharmaceutical Supply Agency of the western cluster.

**Methods:**

An analytical cross-sectional study was conducted between January and February 2020. We selected 269 respondents using a multistage sampling technique. A pretested semi-structured questionnaire was used to collect the intended data. We employed SPSS version 26 and AMOS 22 software to analyze the quantitative data. The findings obtained from the open-ended questions were summarized in word document manually and used to discuss the quantitative data. We tested the hypotheses using structural equation modeling.

**Results:**

Of the 269 questionnaires, 247 were duly completed and returned, making a 92% response rate. The findings indicated that the pre-transaction logistics service components (*β* = 0.31, p < .001), the transaction logistics service components (*β* = 0.54, p < .001), and the post-transaction logistics service components (*β* = 0.62, p < .001) influenced customer satisfaction positively and significantly explaining 77.1% of variations. The respondents indicated that the right person with appropriate qualifications, adequate knowledge, and experience should be employed to provide specific logistics services to please clients.

**Conclusion:**

It is worth pointing out that the higher logistics services performances are likely to enhance customer satisfaction. Therefore, logistics managers should strive to ensure customers get the desired products and services reliably to increase their satisfaction even better than the current performance.

**Supplementary Information:**

The online version contains supplementary material available at 10.1186/s40545-021-00351-6.

## Introduction

Different scholars have recognized that logistics customer service reaps the benefits of customer satisfaction. These studies appreciate logistics service performance as a key feature to achieving value-added services and customer satisfaction [1–3]. Given pharmaceutical demand is a complex process that involves many actors and stakeholders, customer satisfaction can be influenced by several factors. These may range from the ease of placing an order to stock availability and delivery reliability. Pharmaceutical providers can satisfy their customers if they can meet expectations [4–6].

Different researchers disclose that service delivery performance is linked to how logistics service is used as an intermediary operation. Their base of discussion is on how logistics activities enhance product value by accommodating customers’ order requirements. Many researchers demonstrated this by the seven rights of utility creation through logistics services: delivering the right product, at the right quantity, at the right place, in the right condition, at the right time, with the right information, and at the right price [7–10].

Pharmaceutical logistics involves the processes and activities of procurement, warehousing, inventory management, and transport of the products while maintaining stability in medicine quality during inventory management and delivery. It is exceptional compared to other logistics because it must meet the needs of hospitals, clinics, pharmacies, and health centers that take into account storage conditions besides temperature and humidity control [6, 11, 12].

To strengthen the public healthcare supply chain, the Ethiopian pharmaceutical supply agency (EPSA) was established with the mandate of availing affordable and quality pharmaceuticals sustainably to all public health facilities (HFs). Since 2007, the agency is responsible for managing, operating, and developing the public healthcare supply chain in the country. Cognizant of the crucial roles of pharmaceuticals in healthcare delivery, the agency has determined to ensure optimal product availability and excellent customer service by coordinating branches, improving logistics operations, and implementing an agile approach [13, 14].

Despite significant achievements in the public health sector, the logistics process of the agency is yet to create a highly responsive and efficient pharmaceutical logistics system that matches the ever-increasing demand of the public. Concerning this, customers expect more about fill rates and order turnaround time, and the logistics data visibility is still demanding [13]. These expectations may affect customer satisfaction in the logistics service delivery process.

Logistics customer service performance is often examined by dividing its constituent components into three phases to reflect the nature and timing of the particular service delivery as: (1) pre-transaction components that arise before the actual transactions take place (e.g., salesperson quality, method of ordering, system flexibility); (2) transaction components which are directly related to the physical transaction (e.g., stock availability, order cycle-time, order information, delivery reliability, and condition of goods), and (3) post-transaction components involving elements that occur after the delivery have taken place (e.g., repairs, warranties, returns, complaints, invoicing accuracy) [4, 15, 16]. Adjusting the key components of logistics services to customer's expectations is essential at every stage of the service delivery [17].

These days, pharmaceutical customers are the utmost stakeholders in the healthcare supply chain, and ensuring their satisfaction with the logistics services has become worthwhile. The existing researches in the pharmaceutical logistics field focus on inventory levels, facility locations, warehousing, and logistics network designs and have focused on the logistics service providers [18–20]. Most of these studies concentrated on the operational performance of the companies while placing little importance on customers' perceptions. This is an essential gap that paves for studies regarding the logistics service attributes from the customer perspective.

In this study, the researchers sought to modify a logistics service performance scale to obtain customers' perceptions about the agencies customer service agency's performance measures. Hence, the current study attempted to examine the perceived influence of pre-transaction, during-transaction, and post-transaction logistics service performance on customers’ satisfaction in the agencies using the logistics service models contextualized from different kinds of literature. Furthermore, the study intends to determine statistically significant logistics service attributes (LSA) (e.g., personnel contact quality, information quality, timeliness, product availability) from each logistics phase perceived by customers.

## Methods

### Study settings and period

An analytical cross-sectional study was conducted between January and February 2020 among health facilities (HFs) served under the EPSA western cluster. Clustering the branches, a new initiative of the agency, aimed at delivering efficient services by creating mutual coordination among branches on geographic proximity that paves the way to more easily tap performance reports and other information via the seven clusters. The western EPSA cluster consists of Jimma, Nekemte, and Gambella branches. These branches are responsible for supplying pharmaceuticals to affiliated HFs.

### Population and sampling

All HFs located in the western part of Ethiopia, and the staffs of the HFs constituted the source population of the study. The study population included the public HFs grouped under the divisions of the western EPSA, i.e., Jimma, Nekemte, and Gambella. The data sources were employees working at the chosen public HFs and in charge of pharmaceutical logistics management. Respondents who were not available during the data collection time and incomplete or inconsistent data were excluded.

**Fig. 1:**
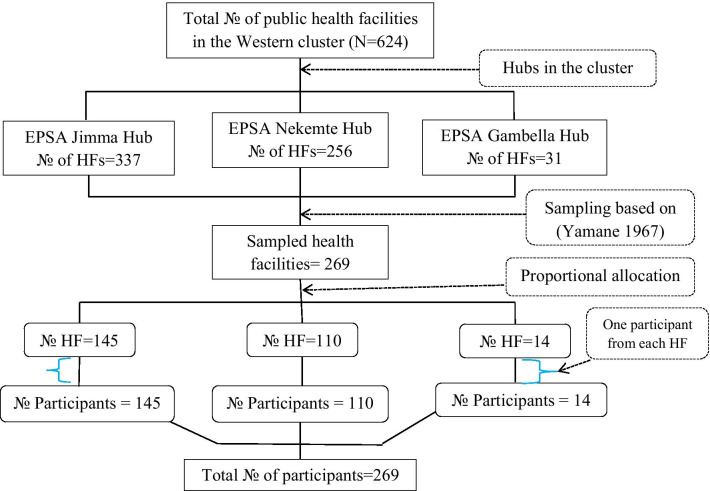
Sampling process of participants ( source: researcher own developed)

We used a multistage sampling technique to select the required number of participants, as shown in Fig. 1. The western EPSA cluster, one of the seven EPSA clusters in Ethiopia, was first selected randomly. Then, we identified and sampled the HFs allocated under the cluster (the western). Lastly, from the sampled HFs, the required number of participants calculated.

There were 624 public HFs grouped under the three hubs. Hence, a total of 244 HFs were estimated using a formula developed by Yamane (1967) by taking a 5% degree of precision [21]. However, the final sample size was 269 HFs, with the addition of 10% for potential non-response. The final sample size (i.e., 269 HFs) was proportionally allocated to the three EPSA hubs. Then, we used a systematic random sampling technique in each hub to select the required HFs. Finally, one respondent from each facility was selected making the sample size of 269. The respondents were health professionals who lead the pharmacy department in their facilities and responsible for pharmaceuticals logistics services.

### Study variables and hypotheses

Through a series of discussions with experienced logistics managers in the branches and a review of previous literature, a total of 35 performance measurement items were identified. Depending on the nature and timing of specific service delivery, the 35 measurement elements were further categorized into nine logistics service attributes (LSA) that can help determine the three processes of the logistics services. Information quality, ordering procedure, personnel contact quality in pre-transaction logistics phase; product availability, order condition, and timeliness at transaction logistics phase, and order accuracy, order discrepancy handling, and complaint handling in the post-transaction logistics service phase (Additional file 1). The following alternative hypotheses (H) were developed and tested.

(H1)Pre-transaction LSP (encompasses Information quality, ordering procedure, and Personnel contact quality) has a significant positive influence on customer satisfaction in the branches.

(H2)During-transaction LSP (operationalized with Product availability, Order condition, and Timeliness) has a significant positive influence on customer satisfaction in the branches.

(H3)Post-transaction LSP (conceptualized to contain Order accuracy, Order discrepancy handling, and complaint handling) has a significant positive influence on customer satisfaction in the branches.

### Data collection tool and procedure

We used a self-administered semi-structured questionnaire to collect the required data. The tool was organized into three parts. The first part dealt with demographic characteristics. The second part contained questions to assess the customers' perceived logistics service attributes. The third part was designed to address the levels of customer satisfaction. We prepared the second and third parts using a five-point agreement and satisfaction Likert scale questions, respectively. Open-ended questions were also used to further explore the perspectives of the customers (Additional file 2). The results were then triangulated with the quantitative findings.

### Data quality assurance

Trained data collectors and supervisors assisted in data collections from HFs affiliated in the same EPSA hubs. The principal investigator gave training to the supervisor and data collectors on the objective of the study and the data collection method. Before the actual study, a pilot test was conducted in the 23 HFs out of the study area. Based on the pilot test, minor adjustments were made to a few of the items to enhance readability. Besides the supervisors, the principal investigator oversaw overall data collection processes. We ensured the technical data quality for its consistency, completeness, and other errors before data entry.

### Data processing and statistical analysis

The data were entered into Epidata software version 3.1 for cleaning and exported to SPSS version 26 and AMOS (Analysis of moment structure) version-22 for analysis. In structural equation modeling, its underlying assumptions that substantially affect the ability to represent multivariate relationships need to be tested [22–24]. In this study, normality, linearity, multicollinearity, and homoscedasticity of the data were tested before conducting the statistical analysis. The outputs of the analyses showed no violation of the underlying assumptions of multivariate statistical analysis. We performed a Pearson’s product-moment correlation (*r*) to measure the strength and direction of the linear relationship between variables. According to a rule of thumb, [0.1 < *r* < 0.3] indicates weak correlation, [0.4 < *r* < 0.6] reveals moderate correlation, and [0.7 < *r* < 0.9] indicative of strong correlation between variables [23, 25].

Exploratory factor analysis (EFA) was conducted to summarize the underlying set of observed variables. We employed a principal component analysis (PCA) with an orthogonal varimax rotation and suppressing all factor coefficients less than 0.4 to enable simple factor loading [22–24, 26]. We checked the appropriateness of data to proceed with EFA by examining the correlation matrix and the KMO (Kaiser–Meyer–Olkin), the measure of sampling adequacy. The use of the orthogonal varimax rotation was also checked because this method assumes components were uncorrelated. Based on the decision rule, an oblique rotation with ‘*Direct Oblimin’* was run to check if the correlation between any two components exceeded 0.32 (i.e., > 10% overlap in variance between components).

To assess goodness-of-data to the model, confirmatory factor analysis (CFA) was performed using the maximum likelihood estimation approach and reconstructed items based on the EFA results [22, 23, 28]. After setting the scale for each LSA’s variance to unity in AMOS software, the relationship between the measured variables and their respective LSA was tested by CFA. We used the usually reported model fit indices of Chi-square (χ2), degree of freedom (*df*), root mean square error of approximation (RMSEA), and Comparative Fit Index (CFI) to test the fit of datasets to the model.

Before examining the structural relationships among latent constructs, the researchers applied multivariate techniques to check the reliability and validity of the measurement model as suggested by [23]. Having identified the determinant items in the LSAs, the constructs have been tested for validity and reliability. Reliability analysis is a technique for assessing the consistency of measured variables by using Cronbach's α statistics and composite reliability (CR) [24, 28]. In this study, we conducted the reliability of variables after EFA by employing Cronbach’s α and composite reliability (CR).

Validity reveals how well a measure reflects its unobservable factors. It is established using relationships between measured items and their constructs, as well as the relationships between constructs. In this study, construct validity was assessed by using CFA in AMOS software. The ability to demonstrate convergence and discriminant validity establishes evidence of construct validity [23, 29, 30]. Convergent validity (CV) means that the variables within a single factor are highly correlated or share a high proportion of variance in common. The CV is usually assessed by high bivariate correlations, significant factor loadings, and the average variance extracted (AVE). The AVE is the sum of all squared standardized factor loadings divided by the number of items. The square of a standardized factor loading represents how much variation in an item is explained by the construct and is termed the variance extracted of the item [22, 23]. Thus, the loading of 0.71 squared equals 0.5. In short, the factor is explaining half the variation in the item with the other half being error variance. Using this logic, an AVE of 0.5 or higher is a good rule of thumb suggesting adequate convergence.

We estimated the discriminant validity to ensure the factors are distinct and did not correlate too loosely with other factors. The rule of thumb is that variables should relate more strongly to their factor than to another factor. Correlations with other constructs below 0.70 are usually accepted as evidence of construct distinctness and thus discriminant validity [22, 23].

Many concepts are inherently latent and cannot be observed directly, hence structural equation modeling (SEM) is particularly beneficial in quantifying these key concepts. SEM is an advanced statistical method for concurrently defining relationships among latent variables while accounting for measurement errors during estimation procedures [22, 23, 31]. Hence, using this model, the measured items were used to estimate the degrees of the latent variables (attributes of the logistics service—first-order factors) that in turn used as indicators for the second-order variables (components of the logistics services) [27, 32].

To test the hypothesis, we used the SEM. The model was run in two phases, with the first phase evaluating the measurement model (instrument validation by CFA). In the second step, we investigated the relationships between the latent variables using path coefficients (*β*). As a final stage, we tested out the hypotheses based on the standardized path coefficients (*β*) and level of significance (p-value).

## Results

### Demographic characteristics of the study participants

Of the questionnaires distributed to 269 respondents, 247 were completed and returned, making a 92% response rate. Most of the participants, 93.1% were from health centers. Male respondents represented 72.1% of the surveyed health facilities. The majority of them, 83.8% were between the ages of 20 and 34 years. Pharmacy professionals represented 65.6% of the respondents. Most of them, 83.4%, had more than two years of work experience, and almost all of the respondents, 93.9% purchased/received products within 6 months (Table 1).

**Table 1:** Demographic characteristics of the study participants

Variables	Frequency	Percentage
Type of health facility		
Health center	230	93.1
Hospital	17	6.9
Gender		
Male	178	72.1
Female	69	27.9
Age in years		
20–24	23	9.3
25–29	104	42.1
30–34	80	32.4
35–39	30	12.1
40–44	10	4.0
Profession		
Pharmacy	162	65.6
Clinical nurse	63	25.5
Laboratory technician	22	8.9
Experience in years		
Less than 2	41	16.6
2 to 4	71	28.7
Greater than 4	135	54.7
Recent purchase or receipt time in months		
Less than 3	182	73.7
3 to 6	50	20.2
7 to 10	10	4.0
Greater than 10	5	2.0

### Correlation analysis

All logistics service attributes (LSA) have a positive significant correlation with customer satisfaction. Most LSAs had a moderate correlation, coefficients ranging from 0.463 to 0.617, with customer satisfaction at p < 0.001 except for ordering procedures and order conditions having values of 0.309 and 0.275 at p < 0.001, respectively. The predictor variables have a positive correlation with each other, with values ranging from 0.05 to 0.567 (Table 2).

**Table 2:** Correlation between study variables

Factors	IQ	OP	PCQ	PA	OC	T	OA	ODH	CH
IQ	1								
OP	.274**	1							
PCQ	.433**	.243**	1						
PA	.284**	.136*	.415**	1					
OC	.200**	.225**	.225**	0.05	1				
T	.308**	.208**	.316**	.273**	.257**	1			
OA	.279**	0.094	.207**	.536**	.185**	.162*	1		
ODH	.371**	.217**	.448**	.331**	.237**	.336**	.141*	1	
CH	.388**	.218**	.567**	.423**	.307**	.297**	.275**	.420**	1
Satis	.492**	.309**	.610**	.617**	.275**	.491**	.463**	.569**	.587**

*IQ* information quality, *OP* ordering procedures, *PCQ* personnel contact quality, *PA* product availability, *OC* order condition, *T* timelines, *OA* order accuracy, *ODH* order discrepancy handling, *CH* compliant handling, *Satis* satisfaction

^**^ p < 0.01, * p < 0.05 (2-tailed)

### Exploratory factor analysis

The correlation component matrix depicted all correlations below the threshold value of 0.32, indicating that the use of varimax rotation was appropriate. The KMO (Kaiser–Meyer–Olkin) measure of 0.828 indicated the appropriateness of data to proceed with EFA. All factors have at least three items with factor loadings from 0.50 to 0.87 that is above the recommended value of 0.5. The eight extracted factors accounted for 68.30% of the total variance in the dataset (Table 3).

**Table 3:** Rotated factor loadings, eigenvalue, and total variance explained

Study variables	Number of items	Factor loadings	Eigenvalue	% of variance explained
Order discrepancy handling (ODH)	4	[0.689–0.788]	7.07	26.16
Personnel contact quality (PCQ)	3	[0.664–0.815]	2.62	9.70
Ordering procedures (OP)	4	[0.652–0.839]	2.12	7.84
Order accuracy (OA)	3	[0.734–0.818]	1.70	6.30
Product availability (PA)	4	[0.651–0.688]	1.49	5.51
Information quality (IQ)	3	[0.640–0.854]	1.43	5.30
Complaint handling (CH)	3	[0.652–0.715]	1.03	3.78
Timeliness (T)	3	[0.497–0.875]	1.01	3.71

Kaiser–Meyer–Olkin (KMO) measure of sampling adequacy = .828

Bartlett's test of sphericity (app. Chi-square = 2846.370, df = 351, and sig. = .000)

Extraction method: principal component analysis

Rotation method: varimax with Kaiser normalization

### Confirmatory factor analysis

**Fig. 2:**
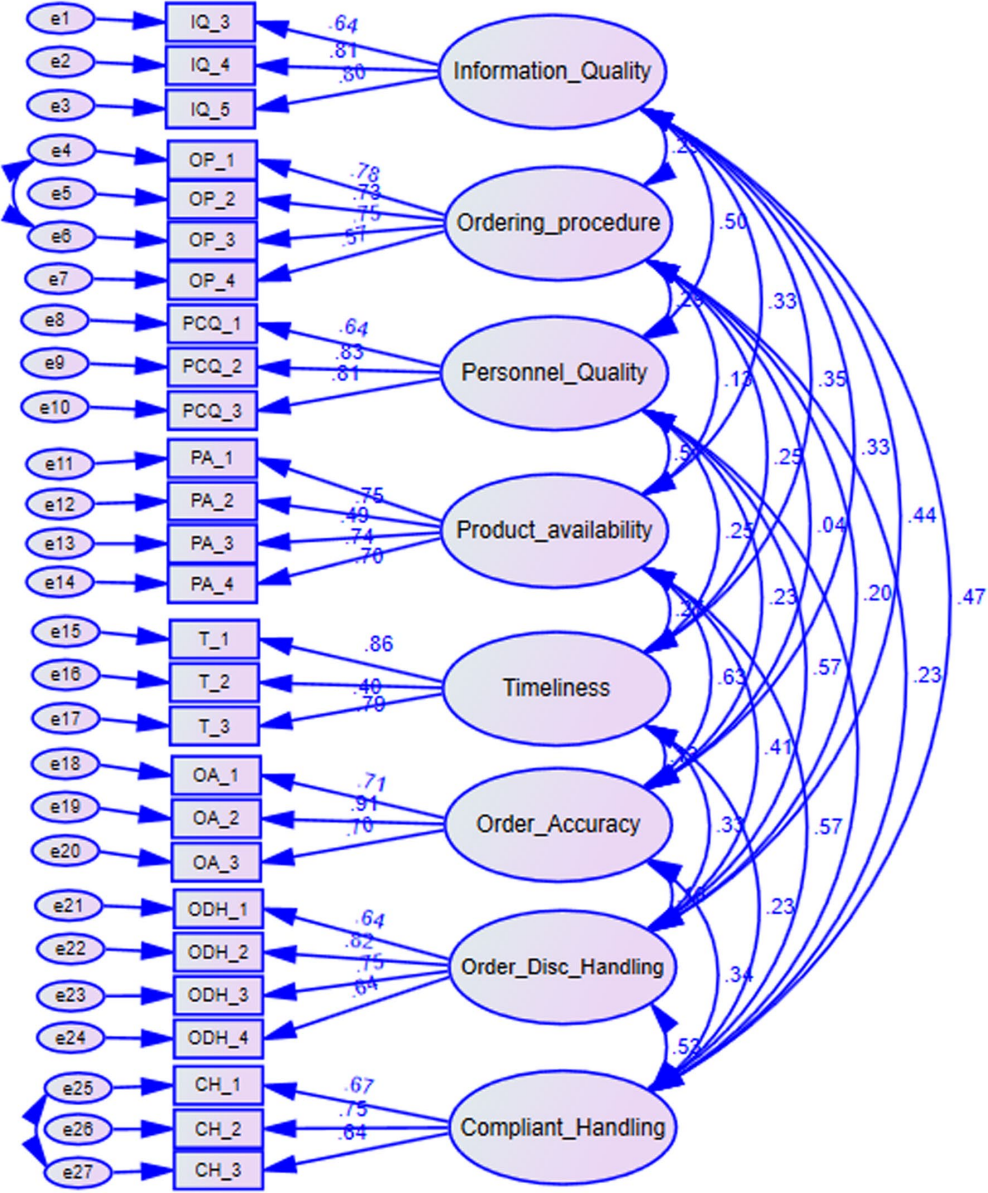
Standardized factor loading of modified items from CFA

The model fit indices of Chi-square (χ^2^) = 500.319, degree of freedom (*df*) = 290 (χ^2^/df = 1.725, p < 0.001), root mean square error of approximation (RMSEA = 0.057), and Comparative Fit Index (CFI = 0.908) was obtained for measurement model. Using the rule of thumb, the results of χ2/df (< 3), CFI (0.908), and RMSEA (0.057) reflect the relatively acceptable fit of the model to the data. The χ2 is significant, which is expected given the sample size (n = 247) (Fig. 2).

### Reliability and validity

As exhibited in Table 4, all LSAs after EFA depicted high Cronbach’s α and CR that exceeded the cut-off value of 0.70, supporting the validity of the measurement scale. On the other hand, convergence validity was confirmed since (a) all pairs of items within components exhibited highly statistically significant (p < 0.01) correlations between them; (b) statistically significant factor loadings (loadings > 0.5) obtained for all items, and (c) the AVE values exceeded the estimates of 0.50 except complaint handling (0.477) and product availability (0.461) which were also close to cut-off points (Table 4).

**Table 4:** Correlation coefficients, discriminant validity, and reliability of factors

Factors	IQ	OP	PCQ	PA	T	OA	ODH	CH	AVE	α	CR
IQ	**0.751**								0.564	0.783	0.793
OP	0.241**	**0.713**							0.508	0.779	0.803
PCQ	0.407**	0.182**	**0.765**						0.585	0.791	0.807
PA	0.270**	0.101	0.400**	**0.679**					0.461	0.754	0.769
T	0.189**	0.239**	0.230**	0.053	**0.719**				0.517	0.708	0.745
OA	0.287**	0.197**	0.304**	0.284**	0.243**	**0.778**			0.605	0.805	0.819
ODH	0.341**	0.127*	0.256**	0.562**	0.223**	0.210**	**0.724**		0.524	0.809	0.808
CH	0.345**	0.198**	0.436**	0.335**	0.221**	0.313**	0.410**	**0.69**	0.477	0.709	0.731
Satis	0.365**	0.191**	0.559**	0.411**	0.303**	0.524**	0.593**	0.606**		0.868	

^**^ p < 0.01, * p < 0.05 (2-tailed), diagonal numbers represent the square root of average variance extracted (AVE) for each factor

*α * Cronbach alpha, *CR* composite reliability

From the CFA output, items significantly loaded on designated components and correlations between the attributes were all less than 0.562, implying all eight factors demonstrated discriminant validity. The AVE estimates greater than the squared correlation between constructs establishes the factor’s distinctness. This implies that the variance explained by its items should exceed than it shares with another construct. As Table 4 depicts, the square root of AVE (*in bold on the diagonal*) is greater than inter-construct correlations, suggesting the discriminant validity of the factors.

### Hypotheses test results

**Fig. 3:**
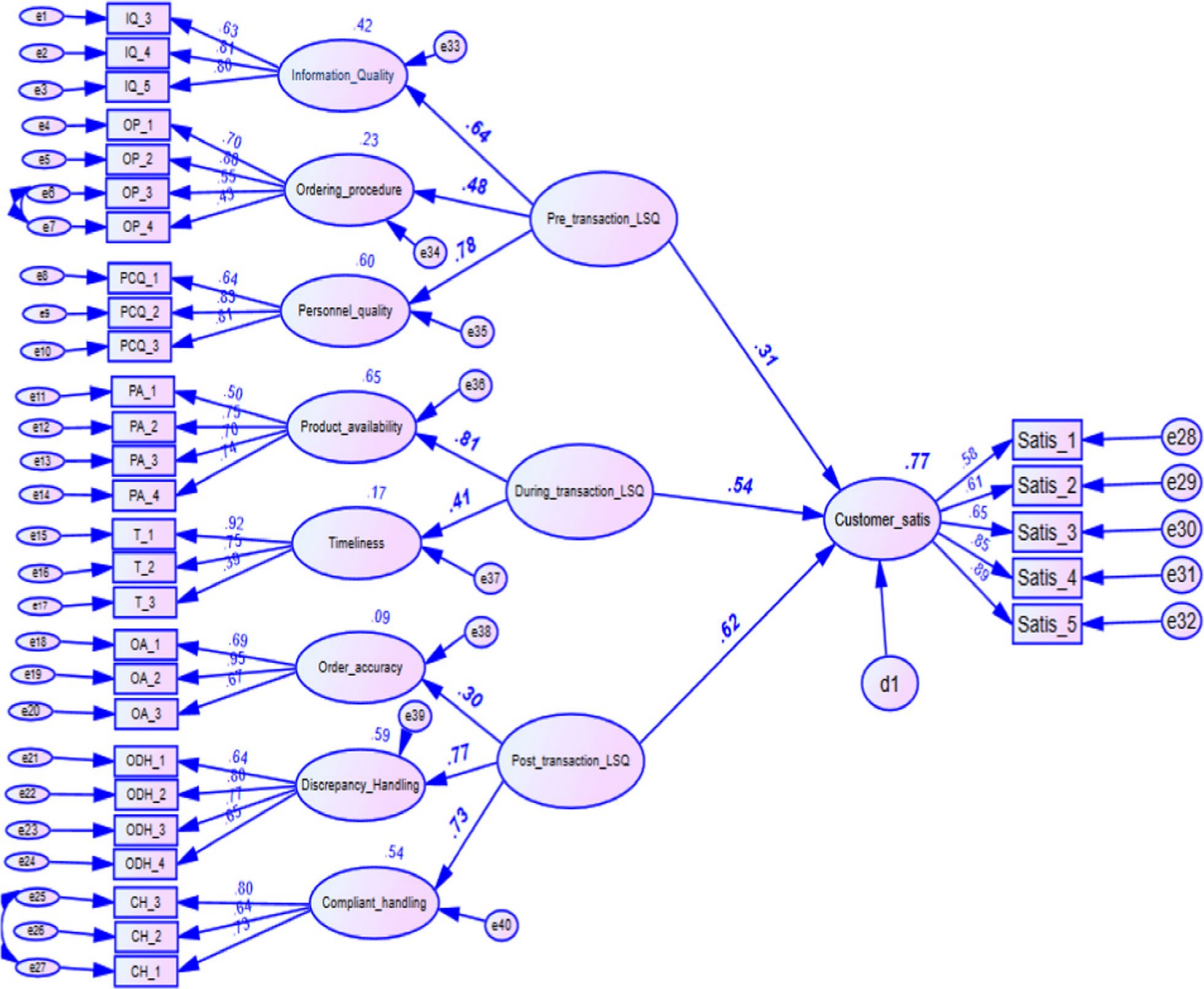
Result of proposed two-step approach structural equation model

Having checked the fit of data for multivariate techniques, the hypotheses were tested simultaneously in an SEM using AMOS. The fit statistics for the structural model are comparable to the measurement model, and demonstrate good model fit (CFI = 0.891 and RMSEA = 0.058) [22, 23, 31]. Investigation of the hypotheses can proceed given an overall fit of data to the model, and the results of the hypotheses tests are provided as follows (Table 5) and ( \* MERGEFORMAT Fig. 3).

**Table 5:** Unstandardized path coefficients—estimate

Path			Estimate	S.E.	C.R.	P
Information Quality	←	Pre-transaction LSP	0.434	0.064	6.760	***
Ordering procedure	←	Pre-transaction LSP	0.328	0.059	5.588	***
Personnel quality	←	Pre-transaction LSP	0.598	0.078	7.687	***
Product availability	←	Transaction LSP	0.534	0.075	7.099	***
Timeliness	←	Transaction LSP	0.289	0.067	4.301	***
Order accuracy	←	Post-transaction LSP	0.245	0.063	3.872	***
Discrepancy handling	←	Post-transaction LSP	0.539	0.060	9.056	***
Complaint handling	←	Post-transaction LSP	0.402	0.064	6.247	***
Customer satisfaction	←	Post-transaction LSP	0.364	0.039	9.259	***
Customer satisfaction	←	Transaction LSP	0.320	0.042	7.526	***
Customer satisfaction	←	Pre-transaction LSP	0.186	0.039	4.812	***

*LSP* logistics service performance, *S.E.* standard error, *C.R. *critical ratio

***  p < 0.001

*H*_*1*_*: Pre-transaction LSP (encompasses Information quality, ordering procedure, and Personnel contact quality) has a significant positive influence on customer satisfaction in the branches*

The results revealed significant positive regression weights for the pre-transaction services at p < 0.001 (i.e., information quality with a standardized coefficient of 0.64, ordering procedure with a coefficient of 0.48, and personnel contact quality with a coefficient of 0.78). The personnel contact quality becomes one of the most important predictors in delivering services as most logistics services involve personnel who often process purchase orders, deliver products, and handle discrepancies. An unstandardized regression weight (estimate = 0.598, p < 0.001) indicated that each additional change (for example moving from 10 to 11 in personnel contact quality) of knowledgeable personnel who give due emphasis to customer’s needs and experienced to perform specific logistics services would result in a 0.598 increase in pre-transaction scores by keeping other predictors constant. According to the analysis, the pre-transaction component had a positive influence on customer satisfaction explaining 31% of variations (*β* = 0.31, p < 0.001).

*H*_*2*_*: During-transaction LSP (operationalized with Product availability, Order condition, and Timeliness) has a significant positive influence on customer satisfaction in the branches.*

The standardized regression weight of product availability and timeliness showed that the attributes predicted the transaction logistics service component positively and significantly with coefficients of 0.81 and 0.41, respectively, at p < 0.001. However, product availability (81%) presents a stronger influence than timeliness (41%). According to the path analysis result, the transaction logistics service component predicted customer satisfaction significantly by 54% (*β* = 0.54, p < 0.001).

*H*_*3*_*:Post-transaction LSP (conceptualized to contain Order accuracy, Order discrepancy handling, and complaint handling) has a significant positive influence on customer satisfaction in the branches.*

The post-transaction logistics service attributes showed a significant positive regression weight at p < 0.001. They included discrepancy handling, complaint handling, and order accuracy with a standardized coefficient of 0.77, 0.73, and 0.30, respectively. The order discrepancy handling has the highest significant contribution of 77% of all attributes included in the analysis, and it accounted for 25.79% of the variations during factor analysis. As a result, the level of customer satisfaction is significantly improved primarily by order discrepancy handling.

The findings indicate that all second-order constructs (pre-transaction logistics service component, during-transaction logistics service component and, post-transaction logistics service component) account for 77.1% of the total variations in customers satisfaction at pharmaceutical supply agencies in Ethiopia (R^2^ = 0.771).

### Results from open-ended questions

The participants disclosed their views, concerns, and suggestions regarding the logistics services offered by EPSA hubs. They indicated that the agencies' customer service at the pre-transaction phase was encouraging. A few respondents, however, stressed the need for experienced druggists for the invoicing of items so that he/she can provide a range of substitute medications.

The findings showed that EPSA also had a strong and weak performance at the logistics services transaction juncture. One of the customers described it as follows:

“*So far, the agency is doing well…… in terms of warehousing, affordability, and delivery reliability. But, not quite enough, as you can perceive stock-outs are increasing day to day. I feel it is becoming hard to handle it. When we see our capacity with limited resources, our hospitals are becoming devastated. Medications are by far costly when purchased from private vendors. Indeed, the agency along with stakeholders should work hard to enhance product availability*.”

Respondents emphasized the need to update the agencies’ pharmaceutical procurement list, creating channels to support product returns, and availing a variety of transport options to provide direct delivery services.

## Discussion

Improving logistics service performance is an ongoing process in the pharmaceutical supply chain [10, 33]. This study aimed to investigate the influence of pharmaceutical logistics service performance on customer satisfaction and the practical relationships between the logistics service attributes in logistics organizations. Based on factor analyses, the study established a reliable and valid conceptualization of logistics service attributes consisting of three second-order dimensions: pre-transaction, transaction, and post-transaction logistics service components. This conceptualization is analogous with technical versus functional logistics service proposed by [34] and operational versus relational order fulfillment services by [18, 35].

As the findings demonstrated, all attributes of the logistics service performance elements require decisive attention to deliver the expected level of satisfaction. The hypothesis testing showed that when customers’ perceptions towards pre-transaction logistics service attributes improved, so does customer satisfaction. The pre-transaction logistics service component—operationalized to contain information quality, ordering procedure, and personnel contact quality showed a positive significant influence on customer satisfaction. This result is consistent with the findings of [8, 36]. The personnel contact quality attribute had the highest significant contribution of 78% to pre-transaction logistics service elements. The agency’s personnel play a key role in receiving orders, communicating order status, and understanding the customers’ expectations of logistics services. Responses from open-ended questions reveal logistics managers need to assign the right employees according to their qualifications, knowledge, and experiences to accomplish specific logistics services to get satisfied customers.

Continuous pharmaceutical availability and reliable delivery promote effective patient care, instigate confidence in the health facility, and contribute to satisfaction among staff [11, 33]. According to the path analyses, the transaction logistics service component had a positive significant effect on customer satisfaction. This emphasizes logistics operations relating to stock availability and timing to focus on the needs of customers to satisfy the services rendered to them. This is in agreement with [8, 36, 37]. Indeed, the more product availability and timely delivery and response, the more satisfying health facilities on the agency’s logistics service performance. According to the open-ended questions, the agency should evaluate ways to improve stock availability and reduce lead times.

Findings show that the post-transaction logistics service component accounted for a 62% variation in customer satisfaction. This result is in line with similar findings from earlier researches [20, 36]. This implies the more complaint and order discrepancy handling as well as order accuracy, the more satisfying health facilities are. Therefore, the hypothesis posited was supported and concluded that the post-transaction logistics service dimension had a positive and significant influence on customer satisfaction. This entails logistics managers to encourage employees to receive feedback on the agency’s core logistics performances.

The results indicated that increased logistics service performance would increase customer satisfaction, while the post-transaction logistics component contributed the most, accounting for 62% of all satisfaction components. These results are comparable with findings reported by [18, 35] while it differs in terms of conceptualization and study setting.

## Conclusion

Our findings indicated that the quality of contact personnel plays a major role in influencing customer satisfaction in pre-transaction logistics service delivery. It can be inferred from the result that customers will be most satisfied when they get products on consistent and dependable delivery from the agency and obtain optimum products and quantities. Indeed, product availability and timeliness have predicted the transaction logistics service component. Order discrepancy handling had the most statistically significant influence of all attributes of logistics service quality in the analysis.

The moderate significant correlations between logistics service attributes showed that the EPSA logistics service processes were moderately correlated. For example, a well-skilled and experienced warehouse operator (personnel contact) processes customers' orders correctly (order accuracy).

With regard to structural equation model results, all logistics service components influenced customers’ satisfaction positively and significantly. Furthermore, the results highlighted that the standardized beta coefficient of the post-transaction logistics service component had the highest value among all research hypotheses. It showed that customers preferred a high degree of satisfaction with post-transaction components followed by transactions and pre-transaction logistics components. In conclusion, it may be worth pointing out that the higher logistics service performance is likely to enhance customer satisfaction (Additional file 2).

### Recommendations

The agency's main features for developing a highly responsive pharmaceutical logistics system that meets the ever-increasing demand for pharmaceutical products are logistics service attributes. To enhance customer satisfaction, the Ethiopian pharmaceutical Supply agency should provide complete, credible, and accurate information; make ordering procedure flexible, convenient, and effective; assign experienced knowledgeable contact person who understands customers’ needs; ensure order accuracy; maintain stock availability; supply products timely as promised; handle order discrepancy satisfactorily, and listen to customer complaints willingly and respond timely. Logistics managers should strive to ensure customers get the desired products and services dependably with a high level of requested quantities with on-time delivery to health facilities to close the existing gaps between customers’ expectations and perceptions.

Therefore, the study recommended that the agency should improve the quality of logistics service attributes to enhance the customers' expectations and perceptions to increase their satisfaction even better than the current performance.

### Limitations

This study investigated the effect of pharmaceutical logistics service performance on customer satisfaction only at the selected branches of the Ethiopian Pharmaceuticals supply agency. Further studies are needed to understand whether the results reported herewith are generalizable across different branches and logistics companies such as wholesaling, retailing, and distributors.

It is also important to note that the results obtained from this study were based on a survey, which captures a situation or an event at a point in time. This shortcoming may be fixed with the data gathered from a more qualitative approach, such as a longitudinal study in future research.

## Supplementary Information

**Additional file 1.** Description of measurement items.

**Additional file 2.** Data collection tools.

## Data Availability

The dataset(s) generated and/or analyzed during the current study are available from the corresponding author on reasonable request.

## Abbreviations

AMOS: Analysis of moment of structures
AVE: Average variance extracted
CR: Composite reliability
CFA: Confirmatory factor analysis
CV: Convergent validity
EFA: Exploratory factor analysis
EPSA: Ethiopian Pharmaceutical Supply Agency
HFs: Health facilities
LSA: Logistics service attributes
LSP: Logistics service performance
PCA: Principal component analysis
SEM: Structural equation modeling
SPSS: Statistical Package for Social Science

## References

[1] Ling TK, Lee CKM (2015). The analysis and case studies of successful express logistics companies. Int J Value Chain Manag..

[2] Rahman NAA, Mohammad MF, Rahim SA, Hassan R, Ahmad MF, Kadir SA (2018). Shipper’s perceptions of aviation Logistics Service Quality (LSQ) of air freight provider. J Eng Appl Sci.

[3] Querin F, Göbl M (2017). An analysis on the impact of Logistics on Customer Service. J Appl Leadersh Manag.

[4] Rushton A, Croucher P, Baker P (2010). The handbook of logistics and distribution management.

[5] Oliver RL (2015). Satisfaction: a behavioral perspective on the consumer.

[6] Chen M-C, Hsu C-L, Lee L-H (2019). Service quality and customer satisfaction in pharmaceutical logistics: an analysis based on Kano model and importance-satisfaction model. Int J Environ Res Public Health.

[7] Vitasek K. Supply Chain Management Terms and Glossary; Council of Supply Chain Management Professionals (CSCMP).2018. p. 117. Available from: https://www.dea.univr.it/documenti/OccorrenzaIns/matdid/matdid982559.pdf. Accessed on 23 July 2020.

[8] Mentzer JT, Flint DJ, Tomas G, Hult M (2001). Logistics service quality as a segment-customized process. J Mark.

[9] Xiang Li. Operations management of logistics and supply chain: issues and directions. J Discrete Dynam Nature Soc. 2014;1-7. 10.1155/2014/701938.

[10] USAID Deliver. The logistics handbook: a practical guide for the supply chain management of health commodities. USAID | DELIVER PROJECT, Task Order 1. 2011. p. 174.

[11] Management Science for Health (2012). MDS-3: managing access to medicines and health technologies.

[12] USAID Deliver. Guidelines for warehousing health commodities, 2nd ed. Arlington: US Agency for International Development; 2014. p. 1–68.

[13] Federal Ministry of Health (FMoH). Ethiopian Health Sector Transformation Plan (2015/16–2019/20). 2015. p. 16–184.

[14] Ethiopian pharmaceuticals Supply Agency (EPSA). Integrated Pharmaceuticals Logistics System (IPLS) in Health Facilities of Ethiopia. 2017. p. 2–124.

[15] Christopher M. Logistics and supply chain management: creating value-adding networks, 4th ed. London: Pearson Education Limited; 2011. p. 1–267.

[16] Kotylak S, Michalowska M, Kulyk P (2017). Assessment of customer satisfaction with logistics service in the light of the results of the research. Management.

[17] Meidutė-Kavaliauskienė AA, Michail L (2014). Consumer satisfaction with the quality of logistics services. Proc Soc Behav Sci.

[18] Alexandre Fun Ghi Su; Mauro Sampaio (2012). The impact of logistics service performance on customer satisfaction and loyalty in Brazilian chemical industry. Int Conf Ind Eng Oper Manag.

[19] Kushwaha S, Sohani N, Kumar S (2017). Effectiveness of logistics, distribution & customer satisfaction in courier company. Int J Sci Res Dev.

[20] Świtała M, Cichosz M, Trzęsiok J (2019). How to achieve customer satisfaction? Perspective of logistics outsourcing performance. LogForum - Sci J Logist.

[21] Yamane T (1967). Statistics: an introductory analysis.

[22] Tabachnick BG, Fidell LS (2013). Using multivariate statistics.

[23] Hair JF, Black WC, Babin BJ, Rolph EA (2014). Multivariate data analysis.

[24] Yockey RD (2018). SPSS demystified: a simple guide and reference.

[25] Gaur AS, Gaur SS (2009). Statistical methods for practice and research.

[26] Samuels P. Advice on Exploratory Factor Analysis. Centre for Academic Success. Birmingham City University . 2016. p. 1–7.

[27] Rahman W, Shah FA, Rasli A (2015). Use of structural equation modeling in social science research. Asian Soc Sci.

[28] Bonett DG, Wright TA (2017). Cronbach's s alpha reliability: interval estimation, hypothesis testing, and sample size planning. J Organ Behav.

[29] Creswell JW (2014). Research design: qualitative, quantitative, and mixed approaches.

[30] Kumar R (2011). Research methodology: a step-by-step guide for beginners.

[31] Kline RB (2015). Principles and practice of structural equation modeling.

[32] Koufteros X, Babbar S, Kaighobadi M (2009). A paradigm for examining second-order factor models employing structural equation modeling. Intern J Prod Econ..

[33] Chopra S, Meindl P (2016). Supply Chain Management: Strategy, Planning, and Operation.

[34] Gronroos C. A service quality model and its marketing implications. Eur J Mark. 1984;8:36–44.

[35] Giovanis AN, Tomaras P, Zondiros D (2013). Suppliers logistics service quality performance and its effect on retailers’ behavioral intentions. Proc Soc Behav Sci..

[36] Giovans AN, Tsoukatos E (2013). On the relationships between logistics service deliverables, customer satisfaction and loyalty in industrial supply chains. J Int Bus Entrep Dev.

[37] Bienstock CC, Mentzer JT, Murphy MB (1997). Measuring physical distribution service quality (PDSQ). J Acad Mark Sci.

