# Supplementary material for: Investigating the effect of pharmaceutical logistics service performance on customer satisfaction: a two-step approach with structural equation modeling
Source: J Pharm Policy Pract. 2021 Aug 2;14:64. doi: 10.1186/s40545-021-00351-6 (PMC8327447; doi:10.1186/s40545-021-00351-6)
Supplement: Supplementary file 1 — Additional file 1. Description of measurement items. [file 40545_2021_351_MOESM1_ESM.docx]

*Table 1. Description of measurement items*

| **Constructs** | **Sub-constructs** | **Measured Items** |
| --- | --- | --- |
| Pre-transaction logistics service | Information Quality | IQ_1: The EPSA communication platforms (website, Viber group or telegram group) provide the most current information  IQ_2: The information about the products or services is complete  IQ_3: The information about the products/services is adequate |
|  |  | IQ_4: The information about the products/services is accurate |
|  |  | IQ_5: The information about the products/services is credible |
|  | Ordering procedures | OP_1: Requisitioning procedures are effective |
|  |  | OP_2: Requisitioning procedures are convenient |
|  |  | OP_3: Requisitioning procedures are flexible (can be sent online) |
|  |  | OP_4: Requisitioning procedures are easy |
|  | Personnel Contact Quality | PCQ_1: Distribution officer makes an effort to understand your needs |
|  |  | PCQ_2: The officer has adequate knowledge to handle your request |
|  |  | PCQ_3: The officer has required experience to process your request  PCQ_4: The employees show real interest in solving a problem when you have |
| During-transaction logistics service | Product availability | PA_1: Ordered products are available in the inventory |
|  |  | PA_2: Ordered quantities are not challenged due to stock shortages |
|  |  | PA_3: Ordered quantities are not challenged due to maximum release quantity |
|  |  | PA_4: Difficulties never occur due to experiences of stock-out items |
|  | Order Condition | OC_1: Products received after order placement are undamaged  OC_2: Orders are packaged conveniently  OC_3: Damages rarely occur as a result of the transportation |
|  | Timeliness | T_1: Ordering and receiving is provided at an appropriate timeframe |
|  |  | T_2: Deliveries reach on the date promised |
|  |  | T_3: The agency gives timely response for emergency/urgent orders |
| Post-transaction logistics service | Order accuracy | OA_1: The products requested delivered, not unordered products |
|  |  | OA_2: The products delivered rarely contain the substituted item |
|  |  | OA_3: The products delivered rarely contain incorrect quantity  OA_4: The Invoices (cash or credit) matches with orders delivered |
|  | Order discrepancy Handling | ODH_1: In a case discrepancy occurred, item return is accepted |
|  |  | ODH_2: Store manager willingly provides exchanges to be replaced |
|  |  | ODH_3: Correction of delivered quality discrepancy is satisfactory |
|  |  | ODH_4: Response to quality discrepancy report is satisfactory |
|  | Compliant Handling | CH_1: The employees are willing to listen to a customer complaint |
|  |  | CH_2: The office/help desk is accessible to handle your complaint |
|  |  | CH_3: The agency is willing to respond to your complaint  CH_4: The agency gives a quick response to your complaint |
| Level of satisfaction |  | SAT_1: How satisfied are you with the EPSA logistics customer services before the actual transaction takes place? |
|  |  | SAT_2: How satisfied are you with the logistics customer services during the actual transaction stage of EPSA? |
|  |  | SAT_3: How satisfied are you with the EPSA logistics customer services after delivery has taken place? |
|  |  | SAT_4: As per your experiences and perceptions, Which word best describes your feelings toward EPSA? |
|  |  | SAT_5: Overall, how satisfied with the EPSA logistics customer service quality? |
