# Supplementary material for: Investigating the effect of pharmaceutical logistics service performance on customer satisfaction: a two-step approach with structural equation modeling
Source: J Pharm Policy Pract. 2021 Aug 2;14:64. doi: 10.1186/s40545-021-00351-6 (PMC8327447; doi:10.1186/s40545-021-00351-6)
Supplement: Supplementary file 2 — Additional file 2. Data collection tools. [file 40545_2021_351_MOESM2_ESM.docx]

***Supplementary file 2. Data collection tools***

***Part I: Background information***

1. Type of Health facility: $\left[ 1 \right]$ Health center $\left[ 2 \right]$ Hospital
2. Gender: $\left[ 1 \right]$ Male $\left[ 2 \right]$ Female
3. Age (in years):$\left\lceil1 \right\rceil$ 20 - 24 $\left[ 2 \right]$25 – 29 $\left[ 3 \right]$ 30 - 34 $\left[ 4 \right]$35-3$9 \left[ 5 \right]$ 40-44 [6] >45
4. Your profession: $\left[ 1 \right]$ Pharmacy $\left[ 2 \right]$Nurse $\left[ 3 \right]$Lab technician $\left[ 4 \right]$others
5. Your experience (in years): $\left[ 1 \right]$ <2 $\left[ 2 \right]$ 2 to 4 $\left[ 3 \right]$ >4
6. How long it has been since you make the last purchase/receipt of products from EPSA?

$\left[ 1 \right]$ <3 months $\left[ 2 \right]$ 3 to 6 months $\left[ 3 \right]$ 7 to 10 months $\left[ 4 \right]$ >10 months

***Part II: Pharmaceutical Logistics service attributes***

Please rate EPSA on the following attributes of the logistics customer services provided to your health facility on a scale of five points with tick “✓”mark where 1= Strongly Disagree, 2= disagree, 3=Neutral, 4= agree and 5= Strongly Agree

| Code | | *Pre-transaction logistics service* | Level of agreement | | | | |
| --- | --- | --- | --- | --- | --- | --- | --- |
|  |  |  | **1** | **2** | **3** | **4** | **5** |
| IQ__1_ | The EPSA communication platforms (website, Viber group or telegram group) provide the most current information | |  |  |  |  |  |
| IQ__2_ | The information about the products or services is complete | |  |  |  |  |  |
| IQ__3_ | The information communicated by the agency is adequate | |  |  |  |  |  |
| IQ__4_ | The information communicated by the agency is accurate | |  |  |  |  |  |
| IQ__5_ | The information communicated by the agency is credible | |  |  |  |  |  |
| OP__1_ | Requisitioning procedures are effective | |  |  |  |  |  |
| OP__2_ | Requisitioning procedures are convenient | |  |  |  |  |  |
| OP__3_ | Requisitioning procedures are flexible (can be sent online) | |  |  |  |  |  |
| OP__4_ | Requisitioning procedures are simple | |  |  |  |  |  |
| PC__1_ | Distribution officer makes an effort to understand your needs | |  |  |  |  |  |
| PC__2_ | The knowledge of the officer to handle your request is adequate | |  |  |  |  |  |
| PC__3_ | The officer has the required product experiences to process your request | |  |  |  |  |  |
| PC__4_ | The employees show real interest in solving a problem when you have | |  |  |  |  |  |
|  | ***During transaction logistics service*** | |  |  |  |  |  |
| PA__1_ | Ordered products are available in the inventory | |  |  |  |  |  |
| PA__2_ | Ordered quantities are not challenged due to stock shortages | |  |  |  |  |  |
| PA__3_ | Ordered quantities are not challenged due to maximum release quantity | |  |  |  |  |  |
| PA__4_ | Difficulties never occur due to experiences of stock out items | |  |  |  |  |  |
| OC__1_ | Products received after order placement are undamaged | |  |  |  |  |  |
| OC__2_ | Orders are packaged conveniently | |  |  |  |  |  |
| OC__3_ | Damages rarely occur as a result of the transportation | |  |  |  |  |  |
| T__1_ | Ordering and receiving is provided at an appropriate timeframe | |  |  |  |  |  |
| T__2_ | Deliveries reach on the date promised | |  |  |  |  |  |
| T__3_ | The agency gives timely response for emergency/urgent orders. | |  |  |  |  |  |
|  | ***Post-transaction logistics service*** | |  |  |  |  |  |
| OA__1_ | The products requested delivered, not unordered products | |  |  |  |  |  |
| OA__2_ | The products delivered rarely contains the substituted item | |  |  |  |  |  |
| OA__3_ | The product delivered rarely contains incorrect quantity | |  |  |  |  |  |
| OA__4_ | The Invoices (cash or credit) matches with orders delivered | |  |  |  |  |  |
| OD__1_ | In a case discrepancy occurred, item return is accepted | |  |  |  |  |  |
| OD__2_ | Store manager willingly provides exchanges to be replaced | |  |  |  |  |  |
| OD__3_ | Correction of delivered quality discrepancies is satisfactory | |  |  |  |  |  |
| OD__4_ | Response to quality discrepancy reports is satisfactory. | |  |  |  |  |  |
| CH__1_ | The employees are willing to listen to your complaint | |  |  |  |  |  |
| CH__2_ | The office/help desk is accessible to handle your complaint | |  |  |  |  |  |
| CH__3_ | The agency is willing to respond to your complaint | |  |  |  |  |  |
| CH__4_ | The agency gives a quick response to your complaint | |  |  |  |  |  |

***Part Three: Level of Customer Satisfaction***

Based on your experiences and perceptions in the Logistics services of EPSA, please put a tick“✓” mark for your level of satisfaction in each statement depicted below (1=Very dissatisfied, 2=Dissatisfied, 3=neutral, 4=Satisfied, and 5=Very satisfied).

| Code | *Statements* | 1 | 2 | 3 | 4 | 5 |
| --- | --- | --- | --- | --- | --- | --- |
| *Sat__1_* | How satisfied are you with the EPSA logistics customer services before the actual transaction takes place? |  |  |  |  |  |
| *Sat__2_* | How satisfied are you with the logistics customer services during the actual transaction stage of EPSA? |  |  |  |  |  |
| *Sat__3_* | How satisfied are you with the EPSA logistics customer services after delivery has taken place? |  |  |  |  |  |
| *Sat__4_* | As per your experiences and perceptions, Which word best describes your feelings toward EPSA? |  |  |  |  |  |
| *Sat__5_* | Overall, how satisfied with the EPSA logistics customer service quality? |  |  |  |  |  |

**Part IV: Open-ended Questions to accommodate additional suggestions**

If you would like to give additional suggestions for pharmaceutical logistics customer services at EPSA, please...

*_______________________________________________________________________________________________________________________________________________________________________________________________________________________________________________________________________________________________________________________________________________________________________________________*

If you could solve one problem with pharmaceutical logistics services, what would it be? *_______________________________________________________________________________________________________________________________________________________________________________________________________________________________________________________________________________________________________________________________________________________________________________________*

***Thank you very much!!!***
